# The South African Rugby Union: SA Rugby Injury and Illness Surveillance and Prevention Project (SARIISPP): Youth Week, Injury Surveillance Report, 2018

**DOI:** 10.17159/2078-516X/2019/v31i1a6365

**Published:** 2019-01-01

## Definitions

### MEDICAL ATTENTION INJURY

The injury definitions were based on the Consensus Statement of 2007 for injury reporting in rugby union.^1^ All injuries that were seen by the Tournament Medical Doctors were classified as Medical Attention injuries, which are defined by the statement as an “*injury that results in a player receiving medical attention”*.^1^

### TIME-LOSS INJURY

Medical Attention injuries were further categorised as Time-Loss injuries, where appropriate, which are defined as “*an injury that results in a player being unable to take a full part in future rugby training or match play*”.^1^

### INJURY RATE

For this report, an injury rate is the number of injuries expressed per 1000 player exposure hours. This normalised version of the number of injuries facilitates comparison between the Youth Week tournaments in 2018, previous tournaments and to other international literature. Moreover, the injury rate is expressed as a mean with 95% confidence intervals. A 95% confidence interval around a mean value indicates that there is a 95% chance (i.e. very high chance) that the true value falls within this range. In this report, we are using the approach of examining the overlap of the confidence intervals to determine whether the injury incidences are significantly different; if the range of confidence interval values of two comparisons do not overlap, there is a strong chance (95%) that their injury rates are different from each other. We have opted for this method because it is conservative and less likely to produce false positive results.^2^

### NEW, SUBSEQUENT AND RECURRENT INJURIES

In 2018, the first injury a player sustained in the tournament, was defined as a ‘*New Injury’*. Any injury that the *same* player sustained after this initial injury was defined as a *‘Subsequent Injury’*. A *‘Recurrent Injury’* was any subsequent injury of the same type and to the same site to that which a player had sustained previously.

## Summary

The BokSmart National Rugby Safety Programme has been exploring the injury rates and patterns at the various SA Rugby Youth Weeks since 2011 as part of the SA Rugby Injury and Illness Surveillance and Prevention Project or SARIISPP. This dataset provides insight into the injury profile of players participating at the youth tournaments, and how the game is changing over time. It also helps to identify any areas of concern that might require further intervention by the governing body to make the game safer. This report focusses on the data from the 2018 boys’ tournaments [u13 Craven week (CWu13), u16 Grant Khomo week (GKu16), u18 Academy week (AWu18) and u18 Craven Week (CWu18)], but also makes comparisons to data collected annually since 2011. The injury data were recorded by the designated data capture researcher assigned to each medical facility at the Youth Week tournaments. In cases where more information became available about an injury after the tournament, the injury data were updated to reflect the most accurate information.

For the 2018 Youth Week tournaments, the Time-Loss injury rates were similar across the tournaments, with the u18 Academy Week having a slightly higher injury rate than the other tournaments. When combining the injury rates of all tournaments from 2011 – 2018 the u18 Craven Week had the lowest injury rate of all tournaments. In 2018, injuries most frequently occurred to the Ball Carrier (i.e. the player being tackled), with the u16 Grant Khomo Week recording the highest injury rates to both the Ball Carriers and Tacklers of all the tournaments. Concussions were the most frequent injury diagnosis recorded in 2018 and although not significant, there was a notable decrease in overall concussion incidence between 2017 and 2018. The concussion incidence increased at the u18 Academy Week and decreased at all the other tournaments. Most new injuries were muscle injuries and the incidence of new injuries was significantly higher than recurrent injuries.

## Injury Incidence

Eighty-two teams competed in the 2018 Youth Week tournaments (CWu13 = 18 teams, GKu16 = 18 teams, AWu18 = 28 teams, CWu18 = 18 teams). There were 236 Medical Attention injuries during all the tournaments in 2018; thirty four percent of these (n = 81) were Time-Loss injuries. The combined tournaments’ injury incidence and 95% confidence intervals for all Medical Attention injuries was 57 injuries (50 to 65)/1000 player hours, and the injury incidence for Time-Loss injuries was 20 injuries (15 to 24)/1000 player hours. There were no significant differences in the incidence of Medical Attention or Time-Loss injuries across the tournaments in 2018 (Table 1).

There were also no significant differences in the number of Medical Attention or Time-Loss injuries per match across the tournaments. From a practical perspective this translates into each tournament requiring similarly scaled medical resources. More specifically, each tournament ideally requires one medical doctor available on-site, per every match played concurrently (Table 2). In 2018 the tournament schedules were structured for the u18 Academy Week and u18 Craven Week tournaments to play at the same venue. Matches were played concurrently. Under these circumstances, two medical doctors are required for sufficient medical support. The u13 Craven Week and u16 Grant Khomo Week tournaments were also played concurrently at the same venue. On days 1, 3 and 4 of the tournaments there were as many as four matches being played simultaneously, while on day 2 there were a maximum of two matches being played simultaneously. A minimum of one additional medical doctor therefore needs to be available on-site on days 1, 3 and 4 of these combined tournaments to provide sufficient medical cover.[Fig f1-2078-516x-31-v31i1a6365]

**Figure 2:**
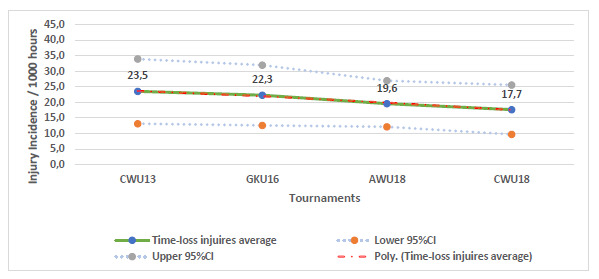
Injury incidence/1000 player hours and 95% confidence intervals of the Youth Week tournaments from 2011 – 2018.

Only Time-Loss injuries were considered for further analyses. When the data were combined (2011 to 2018) there was a tendency for a slight decrease in injury incidence from u13 to u18, with the incidence tending to be lowest at the u18 Craven week tournaments (Figure 2).

## Injury Incidence Trends

### U13 Craven Week

**Figure 3:**
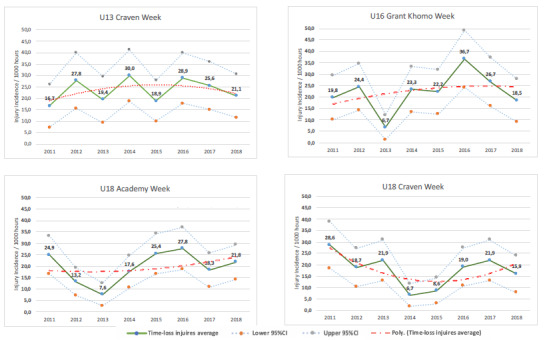
Time-loss Injury incidence for each Youth Week tournament, per year, from 2011 – 2018.

There was an alternating increase-decrease pattern in injury incidence in the u13 Craven Week between 2011 and 2017. In 2018, however, a decrease in injury incidence was observed for the second consecutive year (Figure 3).

### U16 Grant Khomo Week

There was a sizable decrease in injury incidence between 2017 and 2018, with the 2018 injury incidence being the second lowest recorded over the 8 years. The injury incidence decreased for the second consecutive year (Figure 3).

### U18 Academy Week

Following a sizable decrease in injury incidence at the Academy week between 2016 and 2017, there was a slight increase again in 2018 (Figure 3).

### U18 Craven Week

Injury incidence gradually increased each year between 2014 and 2017. Although not significant, a notable decrease in incidence was observed in 2018 (Figure 3). This was the third lowest injury incidence recorded for this tournament.

## Injury Event

When all tournaments in 2018 were combined, injuries most frequently occurred to the Ball Carrier (31%), followed by the Tackler (25%). Ball Carriers had 5 injuries (3 to 7)/1000 player hours and the Tacklers had 4 injuries (2 to 6)/1000 player hours. Injury incidence to both the Tackler and to the Ball Carrier tended to be highest in the u16 Grant Khomo Week (Table 3). When comparing injury rates of Ball Carriers vs. Tacklers across the age-groups, it appears that in 2018, the younger age-groups were more prone to Ball Carrier injuries than to Tackler injuries.

**Figure 4:**
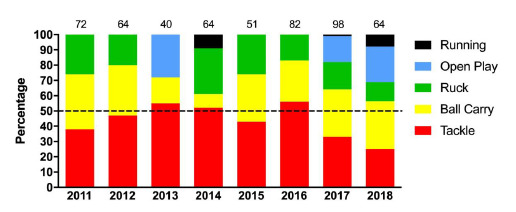
Most common injury causing events in the Youth Week tournaments from 2011 – 2018. Data label number represents the total number of injuries included in the bar graph)

Figure 4 displays the proportion of injuries resulting from the different injury causing events. There was a reduction in the proportion of injuries arising from performing the tackle over the last three years, and an increase in the proportion of injuries from open play in 2018 compared to 2017. A similar pattern was observed from 2016 to 2017 (Figure 4). Of the injuries that occurred in open play in 2018; 7 were in contact events (collisions), and 8 were accidental in nature (slipped, side-stepping, landing). There was no one dominant injury type for injuries occurring in open play.

## Injury Type

**Figure 5:**
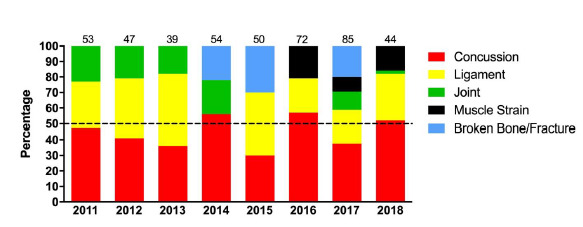
Most common injury types contributing to injury in the Youth Week tournaments from 2011 – 2018. (Data label number represents the total number of injuries included in the bar graph)

In the 2018 tournaments the most common injury type was central/peripheral nervous system (CNS/PNS) injuries, which for all tournaments reflect concussions only (Table 4, Figure 5). There were no significant differences for any of the tournament X injury comparisons. However, the u18 Academy Week did seem to have the highest incidence of CNS/PNS and Joint/Ligament injuries.

## Body Location

In the 2018 tournaments the most common injury location was the Head/Face (30%), with 46% of these Head/Face injuries occurring during the u18 Academy Week.

All the injuries from the 2018 tournaments were grouped according to the four main body location groups (Head & Neck; Trunk; Upper Body; Lower Body); Head & Neck injuries had the highest prevalence (48%) and incidence, 10 injuries (7 to 12)/1000 player hours (Table 5). The u13 Craven Week had the highest tournament incidence of Head and Neck injuries; 13 injuries (6 to 21)/1000 player hours, but this was not significantly different from the other youth weeks.

## New Vs Recurrent

The incidence of *‘New’* injuries in 2018 was 14 injuries (11 to 18)/1000 player hours. The ‘*Recurrent’* injuries were lower at 4 injuries (2 to 6)/1000 player hours. Most of the *‘New’* injuries were to the muscle (75%), while most *‘Recurrent’* injuries were joint injuries (50%).

**Figure 6:**
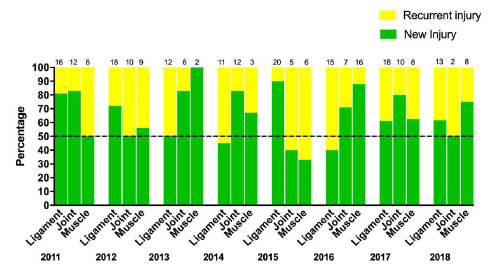
Proportion of New and Recurrent ligament, joint and muscle injuries in the Youth Week tournaments from 2011 – 2018. (Data label number represents the total number of injuries included in the bar graph)

Figure 6 depicts the proportion of *‘New’* and *‘Recurrent’* ligament, joint and muscle injuries across the years. There was an increase in the proportion of *‘Recurrent’* joint injuries from 2017 (20%) to 2018 (50%), while there was a decrease in the proportion of *‘Recurrent’* muscle injuries from 2017 (38%) to 2018 (25%).

## Game Quarter

**Figure 8:**
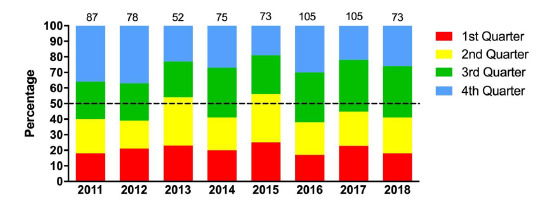
Proportion of injuries occurring each game quarter in the Youth Week tournaments from 2011 – 2018. (Data label number represents the total number of injuries included in bar graph)

Injury occurrence was fairly evenly spread across the match quarters for the 2018 tournaments, with slightly more injuries occurring in the 3^rd^ quarter (33%); 6 injuries (4 to 8)/1000 player hours. There has been no significant change in the distribution of injuries between quarters across the years (Figure 8).

## Concussion

There were 23 concussions in all the tournaments in 2018. This converted to an incidence of 6 injuries (3 to 8)/1000 player hours. The u18 Academy Week had the highest tournament incidence of concussion, 9 injuries (4 to 14)/1000 player hours. Although this was almost 3 times the rate recorded at the u18 Craven Week tournament, this should be interpreted with caution because of the low numbers and wide confidence intervals (Table 6).

**Figure 9:**
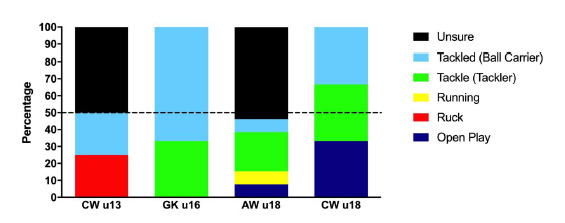
Proportion of concussions caused by the different injury events at the 2018 Youth Week Tournaments (n = 23 concussions).

In the 2018 tournaments concussions to the Tackler (22%) and to the Ball Carrier (22%) were similar (Figure 9).

**Figure 10:**
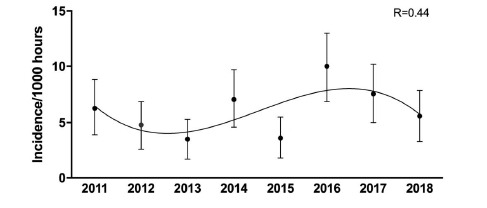
Injury incidence/1000 player hours and 95% confidence intervals of concussions per year at the Youth Week Tournaments from 2011 – 2018.

It appears as if there was an initial slight increase in the rate of concussions up to 2016 and then a gradual decrease. This pattern can be attributed to the interaction of several factors including greater awareness, evolving medical protocols and training techniques, and also to improved sensitivity in recognizing concussions (Figure 10).

**Figure 11:**
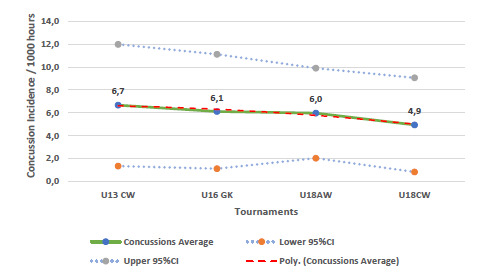
Concussion injury rate/1000 player hours and 95% confidence intervals per Youth Week tournament from 2011 – 2018.

There was a tendency for the combined concussion incidence (2011 – 2018) to decrease as the age of the players increased (Figure 11). This pattern was similar to the pattern of Time-loss injuries (Figure 2).

**Figure 12:**
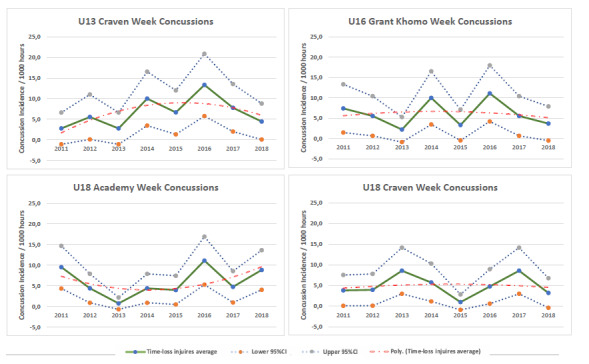
Concussion incidence for each Youth Week tournament, per year, from 2011 – 2018.

The changes in the concussion incidence across tournaments over time fluctuated (Figure 12). For example, the concussions at the u18 Academy Week in 2018 increased from 2017, whereas the U18 Craven Week concussion rate was lower in 2018 compared to 2017. The u18 Craven Week and u18 Academy Week run concurrently and have the same medical support staff. This means that the increase in concussions at the u18 Academy Week in 2018 cannot be attributed to overzealous medical staff. This should be closely monitored in the future.

## Figures and Tables

**Figure 1 f1-2078-516x-31-v31i1a6365:**
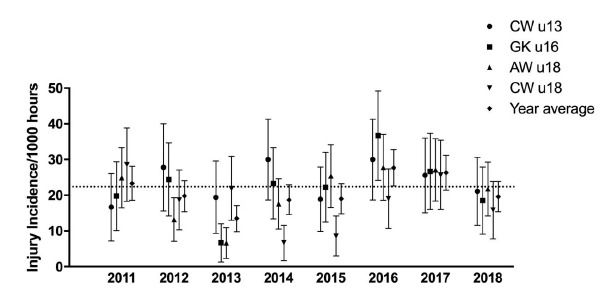
Injury incidence/1000 player hours and 95% confidence intervals of Time-Loss injuries for the Youth Week Tournaments from 2011 – 2018. The dotted line reflects the average incidence for all tournaments over all the included years.

**Table 1:** Number and incidence (95% CI)/1000 player hours of Medical Attention and Time-Loss injuries in the 2018 Youth Week tournaments.

	Medical Attention Injuries		Time-Loss Injuries	
	Number	Incidence	Number	Incidence
CWu13	68	76 (58 – 94)	19	21 (12 – 31)
GKu16	53	65 (48 – 83)	15	19 (9 – 28)
AWu18	67	46 (35 – 57)	32	22 (14 – 29)
CWu18	48	51 (36 – 65)	15	16 (8 – 24)
**Combined Total**	**236**	**57 (50 – 65)**	**81**	**20 (15 – 24)**

**Table 2:** Number of Medical Attention and Time-Loss injuries per match and per hour in the 2018 Youth Week tournaments.

Tournament	Number of matches	Match duration (mins)	Medical Attention injuries/match	Time-Loss injuries/match	Medical Attention injuries/hour	Time-Loss injuries/hour
CWu13	36	50	1.8	0.5	2.3	0.6
GKu16	27	60	2.0	0.6	2.0	0.6
AWu18	42	70	1.6	0.8	1.4	0.7
CWu18	27	70	1.7	0.6	1.5	0.5
**Combined tournament average**	**33**	**63**	**1.8**	**0.6**	**1.8**	**0.6**

**Table 3:** Injury incidence (95% CI)/1000 player hours of Time-Loss injuries to the Tackler and Ball Carrier for the 2018 Youth Week tournaments.

Tournament	Tackler	Ball Carrier
CWu13	2 (1 – 5)	4 (0 – 9)
GKu16	6 (1 – 12)	7 (2 – 13)
AWu18	4 (1 – 7)	5 (1 – 8)
CWu18	3 (0 – 7)	3 (0 – 7)
**Combined Total**	**4 (2 – 6)**	**5 (3 – 7)**

**Table 4:** Injury incidence (95% CI)/1000 player hours of Time-Loss injuries at the 2018 Youth Week tournaments grouped as Joint/Ligament, Muscle/Tendon and CNS/PNS injuries.

Tournament	Joint/Ligament	Muscle/Tendon	CNS/PNS
CWu13	1 (0 – 3)	1 (0 – 3)	4 (0 – 9)
GKu16	1 (0 – 4)	3 (0 – 6)	4 (0 – 8)
AWu18	7 (3 – 11)	3 (0 – 6)	9 (4 – 14)
CWu18	2 (0 – 5)	3 (0 – 7)	3 (0 – 7)
**Combined Total**	**3 (2 – 5)**	**3 (1 – 4)**	**6 (3 – 8)**

**Table 5:** Proportion (%) and incidence (95% CI)/1000 player hours of Time-Loss injuries, grouped by body location, in the 2018 Youth Week tournaments.

	Proportion of injuries (%)	Incidence (95% CI)/1000 player hours
Head & Neck	48	10 (7 – 12)
Trunk	2	1 (0 – 1)
Upper Body	19	4 (2 – 6)
Lower Body	27	5 (3 – 8)

**Table 6:** Number and incidence (95% CI)/1000 player hours of concussions at the 2018 Youth Week tournaments.

Tournament	Number	Incidence
CWu13	4	4 (0 – 9)
GKu16	3	4 (0 – 8)
AWu18	13	9 (4 – 14)
CWu18	3	3 (0 – 7)
**Combined Total**	**23**	**6 (3 – 8)**
